# Retrospective Investigation and Genetic Variation Analysis of Chicken Infectious Anemia in Shandong Province, 2020–2022

**DOI:** 10.3390/vetsci10040263

**Published:** 2023-03-29

**Authors:** Jing Li, Yufei Lou, Peixun Li, Tailong Wang, Zehao Lv, Zhiyun Guo, Ningwei Geng, Fanliang Meng, Sidang Liu, Ning Li

**Affiliations:** 1 College of Animal Science and Technology, Shandong Agricultural University, 61 Daizong Street, Taian 271000, China; lijing19970214@126.com (J.L.);; 2 Shandong Provincial Key Laboratory of Animal Biotechnology and Disease Control and Prevention, 61 Daizong Street, Taian 271000, China; 3 Shandong Provincial Engineering Technology Research Center of Animal Disease Control and Prevention, 61 Daizong Road, Taian 271000, China; 4 Sino-German Cooperative Research Centre for Zoonosis of Animal Origin Shandong Province, 61 Daizong Street, Taian 271000, China

**Keywords:** chicken infectious anemia virus, epidemiological investigation, mixed infection, genotype A

## Abstract

**Simple Summary:**

A total of 854 samples of diseased chickens were collected and tested for chicken infectious anemia virus in chickens between 2020 and 2022. PCR showed 115 positive samples, with a 13.4% positive rate and frequent mixed infections, and 15 representative isolates were selected for VP1 gene sequencing analysis. It was found that most of the CAV in Shandong province was genotype A, and had high homology in strains isolated in recent years. These findings will provide new references for further study of the epidemiology of this disease.

**Abstract:**

Chicken infectious anemia (CIA) is a vertical transmission infectious chicken disease caused by the chicken infectious anemia virus (CAV). The disease can induce stunting and immunosuppression in chicks by infecting bone marrow-derived stem cells, causing huge economic losses for the poultry industry. To determine the prevalence of CIA in Shandong Province, China, 854 suspected CIA samples were collected and analyzed in 13 cities in Shandong from 2020 to 2022. The PCR results showed that a total of 115 CAV were isolated. The CAV-positive rates were 17.21% (26/151) in 2020, 12.23% (35/286) in 2021, and 12.94% (54/417) in 2022, with severe mixed infections. Among them, CAV and fowl adenovirus (FAdV) were the most common, accounting for 40.86%. VP1 gene homology analysis showed that isolated strains shared 96.1–100% homology with the previously reported CAV strains. Genetic variation analysis showed that most of the isolated CAV strains were located in genotype A. These results indicate that CIA infection in Shandong chickens in recent years has been prevalent and mixed infections are common, but there were no significant genetic variations. Our results extend the understanding of the prevalence and genetic evolution of CIA in Shandong Province. They will offer new references for further study of the epidemiology and virus variation and the prevention and control of this disease.

## 1. Introduction

Chicken infectious anemia virus (CAV) is the causative agent of chicken infectious anemia (CIA). This disease is characterized by aplastic anemia and immunosuppression [1]. The predominant natural host of CAV is chickens. CIA is thought to be common in countries that produce chickens, and almost all chicken breeds can be infected by this virus through horizontal and vertical transmission [2]. Chicks up to two weeks old are the most susceptible, and the infected typically exhibit anemia, bone marrow yellowing, and lymphoid organ atrophy [3]. Although CAV infection is usually recessive in older chickens, regardless of the existence or absence of clinical symptoms, it can cause immunosuppression in poultry, reducing the preventive effects of vaccines and increasing the susceptibility to other pathogens [4,5]. This has increased the risk of chicken farming and caused incalculable economic losses to the poultry industry.

CAV belongs to the genus *Gyrovirus* of the family *Anelloviridae* and has no envelope [5,6]. Its genome contains circular single-stranded DNA. Its length is approximately 2 kb, it contains three overlapping open reading frames (ORFs) and encodes three proteins (nucleocapsid protein VP1, auxiliary support protein VP2, and apoptosis-related protein VP3) [6,7,8]. VP1, the principal structural protein of CAV, is related to viral pathogenesis and performs a crucial function in viral transmission and proliferation [6]. During viral growth, VP2 acts as a scaffolding protein to help expose the right epitope conformation of VP1 [8]. VP3 is believed to be a functional protein capable of inducing apoptosis in chicken immune cells [1,8].

CAV was discovered for the first instance in Japan and has since spread to many countries around the world. CAV was first identified and isolated in China in 1996 [9]. After that, CIA spread to many chicken farms in China and became a common infectious disease, affecting the development of the domestic poultry industry. Li et al. collected 1187 clinical samples from chicken farms in various regions of China from 2017–2020 and detected some vertically transmitted diseases. The results also showed that the prevalence of CIA is serious in chicken farms in most parts of China [10]. Shandong Province is one of the most important areas in China for livestock and poultry breeding. The breeding stock of progenitor broilers accounts for 25% of China [11], and it is extremely important to understand epidemiological testing for CIA. In this study, 854 clinical samples of chickens suspected of CAV infection were examined between 2020 and 2022 to determine the prevalence of CIA in Shandong Province. A total of 115 samples were positive, and some representative isolates were selected for genetic evolution analysis based on the VP1 gene. These results could help us learn more about the genetic diversity of CIA.

## 2. Materials and Methods

### 2.1. Sample Collection and Treatment

From 2020 to 2022, 854 samples of diseased chickens were collected throughout Shandong Province. Specifically, cases were obtained from broiler farms, layer farm farms, and breeder chicken farms in Taian city (*n* = 452), Linyi *(n* = 85), Heze (*n* = 68), Jining (*n* = 55), Jinan (*n* = 50), Dezhou (*n* = 37), Liaocheng (*n* = 35), Rizhao (*n* = 25), Weifang (*n* = 21), Zibo (*n* = 12), Binzhou (*n* = 7), Yantai (*n* = 5), and Weihai (*n* = 2). Samples of cases were collected, including bone marrow, liver, spleen, and thymus. At the time of sample collection, all chickens were in sub-healthy condition. Clinical symptoms mainly included depression, weight loss, diarrhea, liver enlargement, pericardial effusion, yellowing of the bone marrow, thymic atrophy, and anemia. All tissue samples were mixed and homogenized in sterile phosphate-buffered saline (PBS) and centrifuged at 12,000 rpm for 5 min; the supernatants were collected and stored at −80°C for virus DNA extraction and detection.

### 2.2. Viral DNA Detection by PCR

The Simply P Virus DNA/RNA Extraction Kit (Bioer, Hangzhou, China) was used to obtain the nucleic acid of each sample, following the manufacturer’s instructions. In our previous study, the PCR primers of CAV, fowl adenovirus (FAdV), reticuloendotheliosis virus (REV), avian leukosis virus (ALV), Marek’s disease virus (MDV), and avian hepatitis E virus (HEV) were reported [12]; primer sequences are shown in Table 1. The total volume of the reaction system was 25 μL. Thermal cycling conditions were as follows: 94 °C pre-denaturation for 30 s, 35 cycles of 98 °C for 10 s, 54~60 °C for 30 s, and 72 °C for 1 min, and the reaction was extended for an additional 5 min before being kept at 4 °C. The results were observed through agarose gel electrophoresis under a gel system imager (Tocan, Shanghai, China).

### 2.3. VP1 Gene Sequencing and Analysis

Some positive PCR samples of CAV were selected and sent to Beijing Genomics Institute (Wuhan, China) for sequencing of the VP1 gene. The sequencing results were analyzed by DNASTAR software (Madison, WI, USA). The partial sequences of the VP1 gene of the CAV isolates generated in this study have been submitted to GenBank under accession numbers (No. OQ240480-OQ420494). Subsequently, the sequences were aligned to reference strains, and homology analysis was performed by Megalign. The MEGAHGFHG X (Mega Limited, Auckland, New Zealand) was used for creating a phylogenetic tree.

## 3. Results

### 3.1. PCR Results and Geographical Distribution of CAV-Positive Samples

The PCR results showed that 115 out of the 854 samples were CAV, with a positive rate of 13.46%. Additionally, 129 samples were FAdV, with a positive rate of 15.1%. Twenty samples were REV (2.34%). ALV was 48, with a 5.62% positive rate. The number of MDV and avian HEV were 46 and 27, respectively.

**Figure 1:**
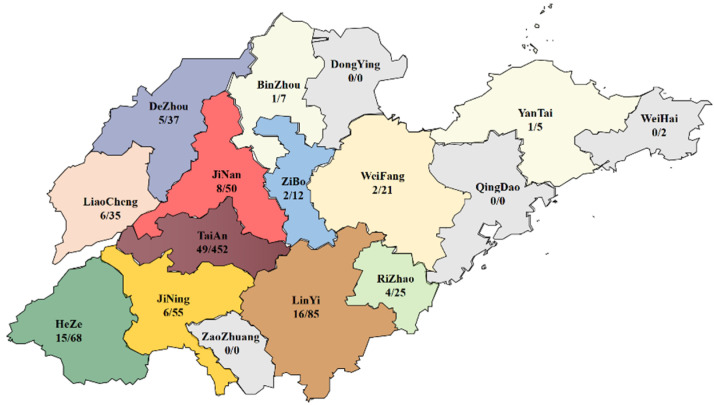
Map of CAV-positive samples distribution in Shandong Province from 2020 to 2022.

The number of CAV-positive samples from different regions in Shandong Province is shown in Figure 1. Heze city had the highest positive rate of 22.05% (15/68), and the lowest positive rate of 9.52% was found in Weifang city.

### 3.2. Mixed Infections of Samples

Among the 115 positive samples for CAV, 47 samples were infected with two or more viruses. Of these, the largest proportion was CAV and FAdV mixed infection, accounting for 60%; 10% was CAV and MDV; 7% was CAV and avian HEV; the proportion of CAV, FAdV, and avian HEV triple infection was 7%; CAV, MDV, and ALV was 4%.

### 3.3. Molecular Characterization of CAV VP1 Gene

In our study, 15 CAV isolates were successfully obtained, and all were submitted to Genbank (No. OQ240480-OQ420494). The lengths of the isolates’ VP1 gene varied from 965–1350 bp, and from these, the full-length gene sequences of two isolates of VP1 were obtained. Sequence analysis revealed that the 15 CAV isolates showed 96.1~100% homology with each other, and the nucleotide sequence homology with the 35 reference strains downloaded from Genbank ranged from 96.1~100%.

We analyzed partial VP1 key amino acid locus substitutions in 15 isolates, as shown in Table 2. All strains had amino acid sites containing glutamine (Q) 141. Methionine (M) was substituted by valine (V) at position 157 in 3 isolates (SDHY-211016, SDHY-210825, and SDHY-220519). Ten isolates (SDHY-210519, SDHY-211016, SDHY-211126, SDHY-211220, SDHY-211224, SDHY-220307, SDHY-220616, SDHY-220801, SDHY-210825, and SDHY-220519) were replaced by serine (S) or alanine (A) or threonine (T) for asparagine (N) at position 287. Proline (P) at position 290 of 4 isolates was replaced by A. The amino acids of 6 isolates mutated from histidine (H) to Q at position 294. In addition, up to 8 amino acid substitutions were found in the isolate SDHY-220519.

**Figure 2:**
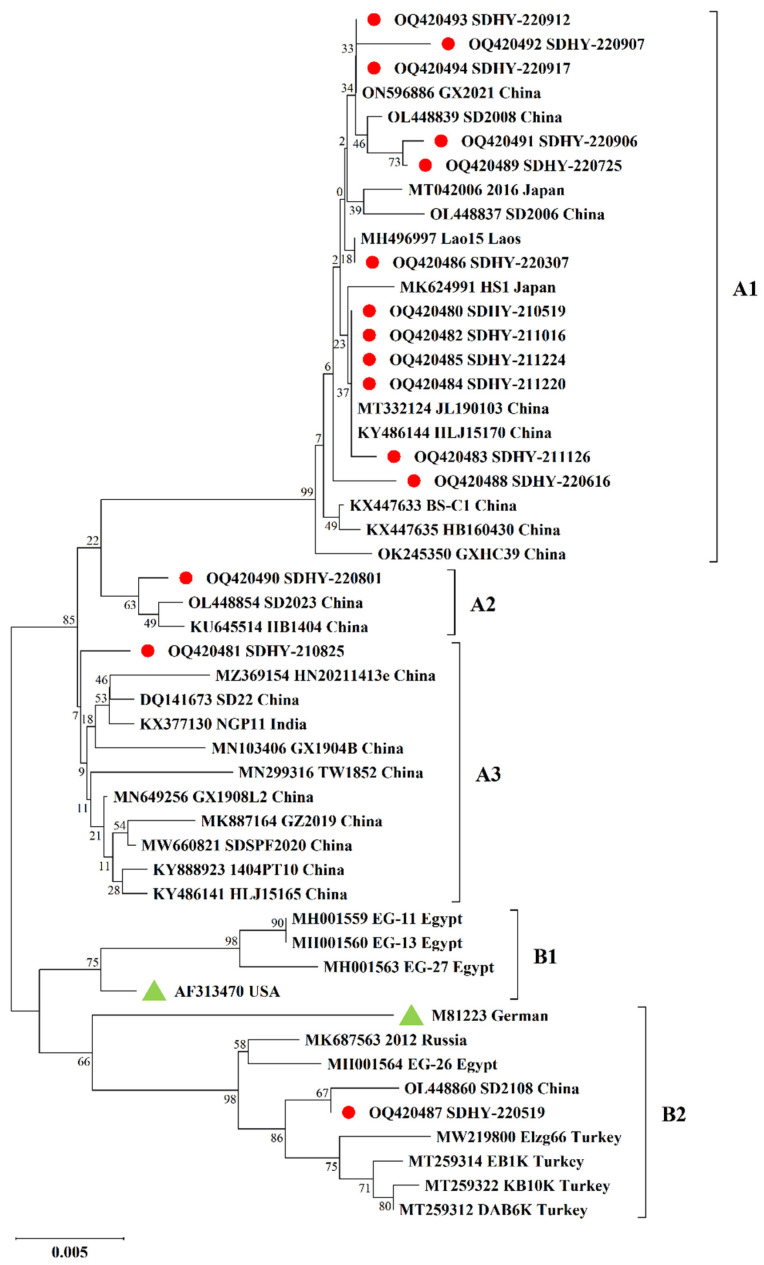
Phylogenetic tree of partial VP1 gene sequences of 15 isolates in this study and referred CAV strains. The phylogenetic analysis was carried out by the Neighbor-joining method by a bootstrap analysis of 1000 replicates using the MEGA X software program. The two major genotypes were identified as A and B. Genotype A was further differentiated into subtypes A1, A2, and A3, whereas genotype B was differentiated into subtypes B1 and B2. The 15 isolates were labeled with red circles, and the attenuated vaccine strain was labeled with a green triangle. A total of 35 reference strains were downloaded from Genbank, isolated from China and other countries.

As in Figure 2, the 15 isolates obtained in this study were compared with 35 representative CAV strains from different countries. Two major genotypes (A and B) were identified by phylogenetic analysis. Genotype A is mostly made up of Asian strains, mainly Chinese isolates, but there are also isolates from Japan, India, and other Asian countries. Genotype B consists mainly of foreign strains such as Egypt, Turkey, American vaccine strain AF313470, and German vaccine strain M81223. Among our isolates, 14 were in genotype A, and SDHY-220519 was in genotype B.

## 4. Discussion

CIA is not a highly lethal avian disease. However, when CAV infects chickens, it suppresses their immunity and leads to vaccine failure and secondary infections, so it is extremely important to investigate CAV prevalence [9,13]. In our study, the total detection rate of CAV in 2020–2022 was 13.46%. Of these, 26 (17.21%) were discovered in 151 examined samples in 2020, 35 (12.23%) in 286 examined samples in 2021, and 54 (12.94%) in 417 samples in 2022. In terms of clinical symptoms, 37 (32.17%) positive samples were yellow bone marrow, 17 (14.78%) positive samples were thymic atrophy, and 13 (11.3%) were anemic, consistent with the clinical signs of a typical CIA [3,4,8]. However, there were still 48 (41.73%) positive samples that did not match the clinical signs of typical CIA. For example, 18 (15.65%) positive samples had abnormal enlargement of the liver, and 11 (9.56%) had pericardial effusion, which may be related to occult infections and mixed infections in CIA [2,9,10]. In the report of Liu et al. [11], CAV was found to be up to 52.05% positive in broilers in Shandong Province, 2020–2021, which was much higher than the results of our study. Notably, the samples were from suspected CAV infections in their study. However, the positive rates of CAV in our study are closer to the 9.4% and 17.14% detection rates discovered during epidemiological surveillance of CIA in Guangxi and Henan Provinces [13,14]. In any case, the results indicate that CIA has been prevalent in Shandong Province in recent years.

CIA is not a new infectious disease, but the detection rate in chickens has been high recently. Previous studies have shown that in the poultry industry, CAV is often mixed with other avian vertically transmitted viruses, such as HEV, FAdV, and ALV. These diseases can cause immunosuppression in chickens and may be an important contributor to the progressive increase in the incidence of CIA [10,15]. In our research, 68 (59.14%) samples were solely infected with CAV, while the remaining 47 samples (40.86%) were mixed infections. Of these, mixed infections of CAV and FAdV were the most frequent at 60%, followed by 10% of CAV and MDV, and 7% of CAV and avian HEV. In addition, triple infections were also found to account for 23% of the cases. Mixed infections of CAV and other viruses were also found in other parts of China. For example, mixed infections of CAV and ALV were found in Guangdong with a 17.5% positive rate, and the positive rate of CAV and FAdV was 26.6% in Guangxi [13,16]. This suggests that mixed infections of CAV are common in China. Whether the interactions between these common immunosuppressive diseases are synergistic or suppressive of infection is unclear; we speculated that these immunosuppressive pathogens may have facilitated the infection of the CAV [2,15].

To determine the molecular characteristics of the CAV isolates widespread in Shandong Province over the past few years, we sequenced and analyzed CAV-positive samples. However, due to the impact of COVID-19, the CAV-positive samples collected in 2020 were not well preserved. Hence, only representative samples in 2021 and 2022 were chosen for sequencing the VP1 gene. Because the VP1 protein has the highest mutation rate, and mutations at certain key amino acid sites can also change the abilities of replication and transmission [13,17], the analysis of key amino acid sites in VP1 proteins was studied. The amino acid substitution patterns of VP1 protein at sites 75, 97, 125, 139, 141, 144, 157, 287, 290, and 294 are shown in Table 1. It is reported that amino acid 287 is the most frequently mutated site [10], and in this article, we also found various types of mutations at this site. All 15 isolates in our study had Q141. The SDHY-220519 isolate had mutations at positions 75 (V to I), 125 (L to I), 139 (K to Q), and 144 (E to Q). Since I75 and Q139 are associated with low pathogenicity of CAV [10,15,17,18,19], this isolate may have a relatively poor capacity for replication and transmission. Q394 can significantly affect the virulence of CAV [8,14,18,19]. The two full-length strains we obtained have position Q394. This result is similar to a previous study where all 33 strains isolated in Shandong Province were Q394, indicating that CAV strains prevalent in Shandong Province may be a relatively highly pathogenic strain [11]. However, the pathogenicity of CAV isolates in chickens still needs further exploration.

In our study, most sequenced CAV isolates were in genotype A, consisting mainly of Asian indigenous strains (14/15). Only SDHY-220519 belongs to the exotic endemic strain consisting of genotype B, suggesting that the spread and inheritance of CAV may have a certain regional characteristic [13,18]. It is noteworthy that this genotype consists mainly of foreign strains, including vaccine strains AF313470 and M81223. This indicates that our isolated strain SDHY-220519 may potentially be used as an attenuated vaccine strain.

## 5. Conclusions

The prevalence of CAV infection has spread in chicken flocks in Shandong Province recently, with serious mixed infections. Genetic evolution showed that CAV strains in Shandong Province were highly pathogenic, but there was no significant change in transmission and replication capacity compared with previous reports. This study will provide a new reference for future CAV epidemiological and viral evolution studies.

## Figures and Tables

**Table 1:** Primer sequences used in the study.

Genes	Primer Sequence (5′-3′)	Tm (°C)	Sizes (bp)
CAV-F	CAGAATTCCCACCTCAAGCGACTTCGAC	55	582
CAV-R	ATGTCGACGGGGCTGAAGGAT		
FAdV-F	AATTTCGACCCCATGACGCGCCAGG	56	508
FAdV-R	TGGCGAAAGGCGTACGGAAGTAAGC		
REV-F	CATACGAGCCAATGGTT	54	300
REV-R	AATGTTGTAGCGAAGTACT		
ALV-J-F	GGATGAGGTGACTAAGA	56	512
ALV-J-R	CGAACCAAAGGTAACACACG		
MDV-F	TCATCAGGGTCTCCCGTCACCT	58	1005
MDV-R	AGAGATGTCTCAGGAGCCAGAG		
HEV-F1	TCGCCT(C)GGTAAT(C)ACA(T)AATGC	60	278
HEV-R1	GCGTTC(G)CCG(C)ACAGGT(C)CGGCC		
HEV-F2	ACA(T)AATGCT(C)AGGGTCACCCG	56	242
HEV-R2	ATGTACTGA(G)CCA(G)CTG(C)GCCGC		

**Table 2:** The analysis of amino acid substitutions in the VP1 proteins of the isolates and the reference strains. The same color in each column means that the amino acids at the locus are identical or the genotypes are identical.

Group	Strain	VP1 Amino Acid Site										Genotype
		75	97	125	139	141	144	157	287	290	294	
1	on596886	V	L	L	K	Q	E	M	N	P	H	A1
2	KU645514	·	M	·	·	·	·	V	·	·	·	A2
3	DQ141673	·	M	·	·	·	·	V	·	·	·	A3
4	AF311470	·	M	I	·	·	·	V	S	A	Q	B1
5	MH001564	I	·	I	Q	·	Q	V	A	·	Q	B2
6	SDHY-210519	·	·	·	·	·	·	·	T	·	·	A1
7	SDHY-211016	·	·	·	·	·	·	V	S	A	Q	A1
8	SDHY-211126	·	·	·	·	·	·	·	T	·	·	A1
9	SDHY-211220	·	·	·	·	·	·	·	T	·	·	A1
10	SDHY-211224	·	·	·	·	·	·	·	T	·	·	A1
11	SDHY-220307	·	·	·	·	·	·	·	S	·	·	A1
12	SDHY-220616	·	·	·	·	·	·	·	T	·	Q	A1
13	SDHY-220725	·	·	·	·	·	·	·	·	·	·	A1
14	SDHY-220906	·	·	·	·	·	·	·	·	·	Q	A1
15	SDHY-220907	·	·	·	·	·	·	·	·	·	·	A1
16	SDHY-220912	·	·	·	·	·	·	·	·	·	·	A1
17	SDHY-220917	·	·	·	·	·	·	·	·	·	·	A1
18	SDHY-220801	·	M	·	·	·	·	·	S	A	Q	A2
19	SDHY-210825	·	·	·	·	·	·	V	S	A	Q	A3
20	SDHY-220519	I	·	I	Q	·	Q	V	A	A	Q	B2

Numbers 1–5 represent the reference strains, and the remaining are isolates in this study. The complete VP1 gene sequences of two isolates (SDHY-210825 and SDHY-220907) were obtained.

## Data Availability

The data that support the findings of this study are available on request from the first author (J.L.) and corresponding author (N.L.).
